# Malaria Pigment Hemozoin Impairs GM-CSF Receptor Expression and Function by 4-Hydroxynonenal

**DOI:** 10.3390/antiox10081259

**Published:** 2021-08-06

**Authors:** Oleksii Skorokhod, Valentina Barrera, Giorgia Mandili, Federica Costanza, Elena Valente, Daniela Ulliers, Evelin Schwarzer

**Affiliations:** 1 Department of Life Sciences and Systems Biology, University of Torino, 10123 Torino, Italy; 2 Department of Oncology, University of Torino, 10126 Torino, Italy; Valentina.Barrera@nhsbt.nhs.uk (V.B.); giorgia.mandili@bioclarma.com (G.M.); federica.costanza676@edu.unito.it (F.C.); elena.valente@unito.it (E.V.); daniela.ulliers@unito.it (D.U.); evelin.schwarzer@unito.it (E.S.); 3 National Health System Blood and Transplant, 14 Estuary Banks, Liverpool GB-L24 8RB, UK

**Keywords:** malaria, hemozoin, 4-hydroxynonenal, monocyte, dendritic cell, granulocyte-macrophage colony-stimulating factor receptor, CD116

## Abstract

Malarial pigment hemozoin (HZ) generates the lipoperoxidation product 4-hydroxynonenal (4-HNE), which is known to cause dysregulation of the immune response in malaria. The inhibition of granulocyte macrophage colony-stimulating factor (GM-CSF)-dependent differentiation of dendritic cells (DC) by HZ and 4-HNE was previously described in vitro, and the GM-CSF receptor (GM-CSF R) was hypothesised to be a primary target of 4-HNE in monocytes. In this study, we show the functional impact of HZ on GM-CSF R in monocytes and monocyte-derived DC by (i) impairing GM-CSF binding by 50 ± 9% and 65 ± 14%, respectively (*n* = 3 for both cell types); (ii) decreasing the expression of GM-CSF R functional subunit (CD116) on monocyte’s surface by 36 ± 11% (*n* = 6) and in cell lysate by 58 ± 16% (*n* = 3); and (iii) binding of 4-HNE to distinct amino acid residues on CD116. The data suggest that defective DC differentiation in malaria is caused by GM-CSF R dysregulation and GM-CSF R modification by lipoperoxidation product 4-HNE via direct interaction with its CD116 subunit.

## 1. Introduction

Malaria is still the most important life-threatening parasitic disease according to the World Health Organisation report, with an estimated 229 million cases and 409,000 deaths occurring in 2019. The immune response during malaria is complex, and immune depression has been described as an important malaria complication [1,2,3,4,5]. Mechanisms and pathomechanistic factors involved in imbalanced immune response are partly known, but many players still need to be investigated.

Studies in malaria models and patients revealed a role of malaria pigment hemozoin (HZ) as pathomechanistic factor in malaria. The nanocrystal (core) of HZ is formed from hemoglobin-heme by the malaria parasite during its proliferation in the infected host red blood cell (RBC). The appearance of HZ in circulating monocytes [6,7] is associated with severe malaria. HZ can non-enzymatically induce lipoperoxidation [8,9,10] and inhibits the immune response in established animal models [11,12]. Human monocytes are strongly activated by their first HZ contact, avidly phagocytose HZ [13,14], and produce pro-inflammatory cytokines and ROS. This initial hyperactive state of the monocytic cells is transient and replaced after few hours by the permanent inhibition of various important immune cell functions, such as motility [15], respiratory (oxidative) burst, further phagocytosis cycles [16], antigen presentation, and the ability to differentiate to macrophages or dendritic cells (DC) [14,17,18,19,20] caused by the persistence of HZ in lysosomes. Although still under investigation, HZ-elicited lipoperoxidation can be considered as a powerful pathomechanism in malaria [9,21].

Oxidative stress and lipoperoxidation play important roles in malaria pathogenesis [21,22,23,24,25]. Pro-oxidants and oxidatively modified proteins strongly increased in the plasma of malaria patients [26,27], and lipoperoxidation products such as thiobarbituric acid-reactive substances or 4-hydroxynonenal (4-HNE) were found in malaria patients’ RBC [28,29]. 4-HNE is considered an oxidative stress marker in various diseases [30,31], including malaria [29]. It is important to note that HZ carries especially high doses, approximately 50 µM, of 4-HNE cargo into monocytes during phagocytosis [32], and, importantly, 4-HNE is further produced during HZ persistence in phagocytes as undigested product [9]. The presence of substantial amounts of bioactive 4-HNE in monocytes explains the sustained interest in 4-HNE as a pathomechanistic factor. 4-HNE was shown to play a role as a regulatory signal molecule [33,34], and increased 4-HNE levels impaired key stem cell receptor functions during in vitro erythropoiesis [35]. Here, we extend our former studies to immune receptors and focus on GM-CSF R as likely target for 4-HNE in monocytes. GM-CSF is one of the most important cytokines in humans for regulating the immune defence, as well as priming monocytes and granulocytes for respiratory burst [36,37], and it is crucial for proliferation, differentiation, and maturation of myeloid cells [38]. The receptor for GM-CSF (GM-CSF R) is comprised of two subunits, α and β, whose association is increased by GM-CSF binding [38,39]. The ligand-specific subunit α (CD116, CSF2RA) includes a large extracellular domain with fibronectin type-III beta barrel structures, a single transmembrane domain, and a small cytoplasmic domain for interaction with the β subunit (CD131), the latter consisting of a more extensive intracellular domain with multiple tyrosine residues that are targets for phosphorylation [40]. After GM-CSF binding and assembly of the high affinity GM-CSF R, tyrosine kinases such as JAK2 are recruited to the intracellular domain of the β subunit to activate the JAK/signal transducer and activator of transcription (STAT), ras/mitogen-activated protein kinase (MAPK), and phosphatidylinositol 3-kinase (PI3-kinase) pathways, leading to increased proliferation; maturation; survival; and activation of myeloid lineages, monocytes, and monocyte-derived cells [38,40]. Structural and functional consequences of oxidative stress for GM-CSF R are little studied, but the importance of this subject may be deduced from several clinical studies that describe changes in GM-CSF/GM-CSF R in inflammatory diseases, almost always accompanied by strong oxidative imbalances [38,41]. The impairment of GM-CSF-dependent differentiation of DC from monocytes by 4-HNE and 4-HNE-producing HZ, which is permanently stored in lysosomes after its phagocytosis [20,32], led us to study GM-CSF R as target for 4-HNE and resulting molecular modifications as causes for its malfunction. The capacity of 4-HNE to covalently bind accessible cysteine, lysine, histidine [30], and occasionally arginine [42] residues in proteins might result in direct structural modification of GM-CSF R by 4-HNE or interfere with surface expression of the receptor in cells with elevated level of lipoperoxidation.

Here, we show changes in GM-CSF R expression and posttranslational modifications of the receptor by 4-HNE, which both may explain the impaired DC differentiation in HZ-laden or 4-HNE-treated precursor cells. The observed modifications of GM-CSF R may contribute to gain insight into the inadequate immune response described in malaria.

## 2. Methods

### 2.1. Reagents

Unless otherwise stated, reagents were purchased from Merck Sigma Aldrich (St. Louis, MI, USA). Cell culture supplements were obtained from Invitrogen Thermo Fisher Scientific (Waltham, MA, USA).

### 2.2. Culturing of Plasmodium falciparum (Pf) and Isolation of HZ

This study was carried out in accordance with the Declaration of Helsinki and authorised by the local Ethical Committee. Blood from healthy adult donors of both sexes was obtained from the local blood bank (AVIS, Torino, Italy), treated with heparin, and promptly used for RBC isolation. *Pf* parasites (Palo Alto strain, mycoplasma-free) were kept in culture with donor RBC as described [35]. HZ was isolated from cultures during the first 2 days after infection of RBC added to schizonts (multinucleated parasite form). After 5000× *g* on a discontinuous Percoll-mannitol density gradient, HZ was collected from the 20/40% and trophozoites and schizonts from the 40/80% interphase. HZ was washed five times with 10 mM HEPES (pH 8.0) containing 10 mM mannitol at 4 °C and once with phosphate-buffered saline (PBS), and stored at 20% (*v*/*v*) in PBS at −20 °C.

### 2.3. Opsonisation of HZ, Latex Beads, and RBC

HZ was thawed, washed once, and finely dispersed in PBS at 30% (*v*/*v*), and latex beads (0.114 µm diameter) suspended at 5% (*v*/*v*) in RPMI 1640 were added separately to the same volume of fresh human AB serum and incubated for 30 min at 37 °C for opsonisation.

Freshly drawn, PBS-washed RBC (control cells) suspended at 50% hematocrit in PBS, supplemented with 10 mM glucose (PBS-G), were incubated with half the volume of human anti-D IgG (Partobulin; Baxter, Deerfield, MA, USA) for 30 min at 37 °C for opsonisation, and washed three times with PBS-G.

### 2.4. Isolation of Monocytes, Phagocytosis of Opsonised HZ, RBC, Latex Beads, and Treatment with 4-HNE

**Figure 1:**
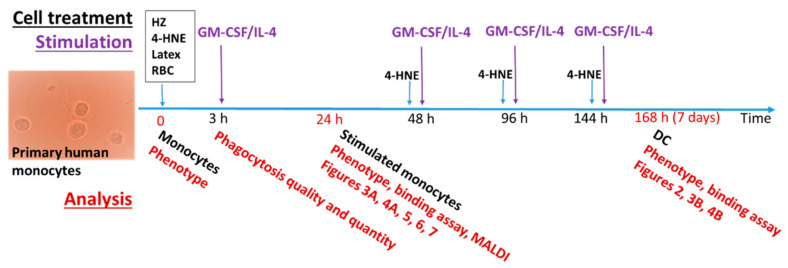
Scheme of experimental design. Experiment was started when adherent human monocytes were supplemented with hemozoin (HZ), 4-hydoxynonenal (4-HNE), latex beads (Latex), or RBC. The phagocytosis was completed in 3 h, when non-phagocytosed material was washed out. Monocytes were differentiated to DC during 7 days by supplementation with GM-CSF and IL-4 every 48 h. 4-HNE and GM-CSF/Il-4 were repeatedly added after culture medium changes, as shown in the diagram. Performed analysis and figures which summarise obtained data are indicated at respective points below the timeline.

The timeline for cell treatment and analysis is summarised in Figure 1.

Monocytes were isolated from freshly drawn peripheral blood of healthy human donors (AVIS, Torino, Italy) by Ficoll centrifugation and lymphocyte depletion with PanT/PanB-Dynal beads (Dynal, Oslo, Norway), as described previously [18]. Two millilitres of monocytes, resuspended at 10^6^ cells per millilitre of RPMI 1640 cell culture medium supplemented with l-glutamine, sodium pyruvate, penicillin/streptomycin, nonessential amino acids (RS), and 1% (*v*/*v*) FCS (RS-FCS medium), were plated in each 35 mm diameter culture dish. After a 30 min incubation at 37 °C, dishes were washed three times with RPMI 1640 to remove non-adherent cells. Two millilitres of RS supplemented with 5% (*v*/*v*) human AB-serum (EuroClone, Wetherby, UK; RS-HS medium) was added to each dish. Phagocytosis and cell treatment were started at time 0 by adding either opsonised HZ (50 RBC equivalents, in terms of heme content, per monocyte), RBC (50 cells per monocyte), latex beads (10 µL of a 100-fold dilution of the opsonised latex bead suspension per 10^6^ monocytes), or 4-HNE at 1 µM final concentration to adherent monocytes. The addition of 4-HNE at 1 µM final concentration was repeated each second day after cell culture medium replacement before cytokine supplementation, unless otherwise noted. In some experiments, 10 µM 4-HNE was added once as bolus at time 0. 4-HNE was freshly activated from (*E*)-4-hydroxynonenal-dimethylacetal, according to manufacturer protocol, immediately before being added to cells. Untreated monocytes were kept in parallel as control under the same conditions. Dishes with control monocytes, HZ-, RBC-, latex-fed, and 4-HNE-treated monocytes were prepared in triplicate.

After a 3 h incubation, phagocytosis was stopped by three washings with RPMI 1640, and phagocytosis was assessed in one of the replicate dishes with RBC- and HZ-fed cells and compared to controls. Two replicate dishes with monocytes of any treatment were further incubated for differentiation to DC (see below) and analysis after 24 h and 7 days.

HZ and RBC phagocytosis by monocytes was quantified by luminescence, as described previously [43], and visually confirmed by microscopy in bright field (Leica DR IRB fluorescence microscope equipped with a Leica DFC 420C camera, a 63× oil planar apochromatic objective with 1.32 numerical aperture, version 3.3.1 of the Leica DFC image software (Leica Microsystems, Wetzlar, Germany)).

### 2.5. DC Differentiation

Adherent washed control monocytes, HZ-, RBC-, latex bead-fed, or 4-HNE-treated monocytes were supplemented with 80 ng/mL GM-CSF and 40 ng/mL IL-4 (R&D) in RS-HS medium for differentiation as shown in the timeline (Figure 1). At the second, fourth, and sixth days of cell differentiation, half of the culture medium was replaced, keeping attention on not removing semi-adherent differentiating DC. Fresh medium was supplemented with GM-CSF and IL-4 at final concentrations of 80 ng/mL and 40 ng/mL, respectively.

### 2.6. Flow Cytometry Analysis of Cell Phenotype, CD116 Expression, and Surface 4-HNE-Protein Conjugates

Monocyte and DC phenotypes were characterised in terms of CD1a, CD14, CD40, CD83, CD87, MHC class II (all antibodies were from BD Biosciences, NJ, USA), or MHC class I (W6/32 hybridoma; American Type Culture Collection, Manassas, VA, USA) antigen expression by flow cytometry (FACS; FACS Calibur cytofluorograph, BD Biosciences) before monocyte treatment at time 0, then at 24 h after starting phagocytosis and at day 7 of differentiation to DC (Figure 1). The surface antigen expression was measured as mean fluorescence intensity (MFI) and values for treated cells were referred to the value of untreated control cells.

CD116 expression was assessed by FITC-conjugated mouse anti-human CD116 antibody (Clone M5D12, BD Pharmingen, San Diego, CA, USA) [44] in human monocytes at 24 h after the start of treatment and consequent stimulation with GM-CSF/IL4 for differentiation.

Conjugates of 4-HNE with cell surface antigens were quantified by anti-4-HNE-conjugate antibody (clone HNEJ-2, Abcam, Cambridge, UK) [45,46] in stimulated monocytes and monocyte-derived DC at 24 h and 7 d, respectively, after treatments and GM-CSF/IL4 supplementation. For 4-HNE conjugate assessment, monocytes were washed in PBS, supplemented with bovine serum albumin at 1% (*w*/*v*), and incubated with saturating concentrations of anti-4-HNE monoclonal mouse primary antibody (1:50 dilution) at room temperature for 1 h. After three washes, bound antibodies were revealed by FITC-conjugated anti-mouse IgG secondary antibody (1:300 dilution). MFI was measured and data were analysed using a FACS Calibur cytofluorograph (Becton–Dickinson, Franklin Lakes, NJ, USA) and CellQuest software (Becton–Dickinson).

### 2.7. Fluorescent GM-CSF Binding to Cells

For functional characterisation of GM-CSF R, FITC-conjugated GM-CSF was supplemented instead of unconjugated reagent, on the basis of the manufacturer’s indications (Invitrogen, Thermo Fisher Scientific). Ligand binding was assessed as MFI, and data were analysed using a FACS Calibur cytofluorograph (Becton–Dickinson, Franklin Lakes, NJ, USA).

### 2.8. Whole Cell Lysate Analysis for CD116 Expression: Sodium Dodecyl Sulfate Polyacrylamide Gel Electrophoresis (SDS-PAGE) and Western Blotting

Adherent cells were washed with ice-cold PBS-G, containing 5 mM mannitol (final concentration) and Complete protease inhibitor cocktail (Roche Diagnostics, Indianapolis, IN, USA), and lysed with Laemmli sample buffer in the tissue culture dish to avoid artefacts by cell harvesting. The quantity of lysate proteins was determined by the Bradford protein assay (Bio-Rad, Hercules, CA, USA). Equal amounts of proteins of different lysate samples were separated by SDS-PAGE and transferred to nitrocellulose membranes by Western blotting using standard protocols (Bio-Rad). Transfer efficiency and protein amount in each lane were checked by Ponceau red staining of the nitrocellulose membrane. CD116 presence in the lysate of different samples was detected by anti-CD 116 (R&D Systems, Minneapolis, MN, USA) and visualised by horseradish peroxidase-conjugated secondary anti-mouse antibody and enhanced chemiluminescence (ECL) assay (Bio-Rad). To guaranty the comparison of same cell numbers, we detected actin as housekeeping protein by anti-actin C-2 monoclonal mouse antibody (Santa Cruz Biotechnology; Santa Cruz, CA, USA), and visualised it by secondary anti-mouse antibody and ECL as specified for CD116. The arbitrary optical density of labelled bands was acquired and compared using ImageLab software version 4.1 (Bio-Rad).

### 2.9. Identification of 4-HNE Binding Sites in GM-CSF by Mass Spectrometry

Protein bands localised by anti-CD116 antibodies after Western blotting were excised from a duplicate gel and subjected to in-gel tryptic digestion and mass spectrometry analysis, as described previously [15]. Proteins were identified by peptide mass fingerprinting against the UniProt sequence database, introducing 4-HNE as a variable modification from the default Mascot software list of modifications (Matrix Science, London, UK). Further search parameters included taxa Homo sapiens, trypsin digest, protein molecular mass, monoisotopic peptide masses, two missed cleavages by trypsin, and peptide mass deviation tolerance of 0.5 Da. Identification of protein bands was obtained from triplicate analysis.

### 2.10. Apoptosis Analysis

Apoptosis was tested by FACS after annexin V–FITC staining, following the manufacturer’s specifications (Apoptosis Detection Kit; Merck Sigma Aldrich).

### 2.11. Statistical Analysis

The values from at least three independent biological replicates are presented as the mean ± SE. Statistical significance (*p*-value) was calculated by non-parametric Mann–Whitney test. For the significance test: * *p* < 0.01, ** *p* < 0.05.

## 3. Results

### 3.1. HZ and 4-HNE Impaired the Differentiation of Monocytes to DC

Previously described phagocytosis of HZ, neutral latex beads, and RBC by monocytes was confirmed by microscopic examination and biochemical luminescence analysis (data not shown). While RBC were digested and not seen after 24 h, HZ and latex beads remained visible inside differentiating monocytes in initial quantity for the whole period of observation (up to 7 days) confirming the persistent presence of ingested HZ (approximately 20 fmole heme for monocyte).

**Figure 2:**
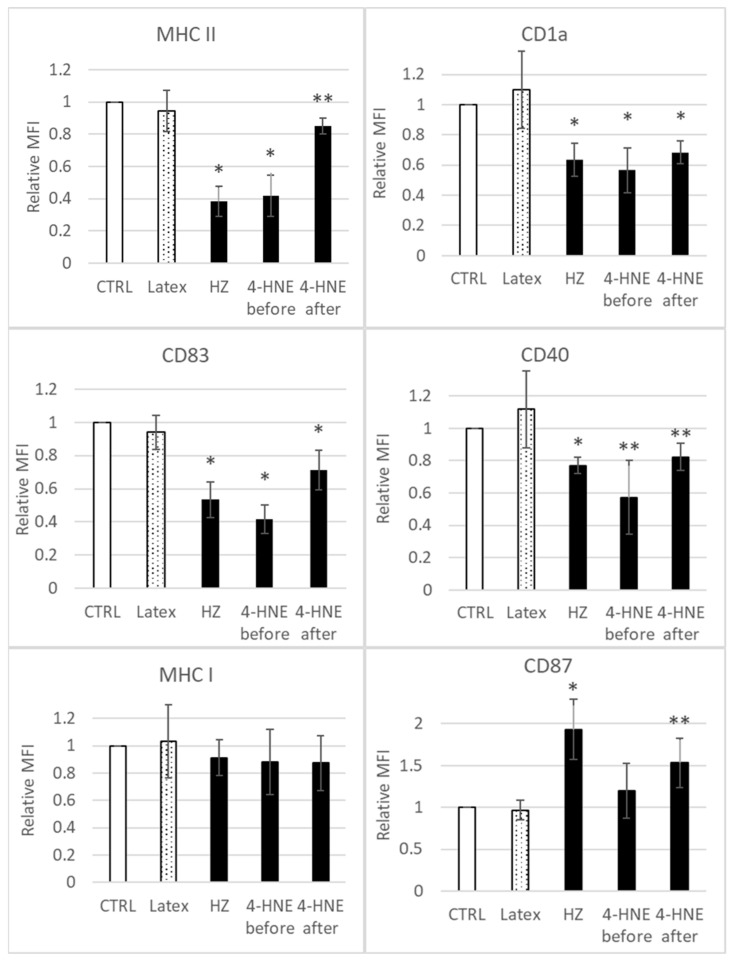
HZ phagocytosis and 4-HNE treatment affected the expression of DC markers. Surface antigen expression in monocyte-derived DC, which had phagocytosed neutral latex beads (Latex), hemozoin (HZ) or were repetitively treated with 1 µM 4-HNE during differentiation either before (4-HNE before) or after (4-HNE after) GM-CSF/IL-4 stimulation, which was quantified by flow cytometry after immunostaining at day 7 of differentiation and related to MFI in untreated control DC (CTRL). Mean values ± SE. Significance: *, *p* < 0.01; **, *p* < 0.05, *n* = 3 donors.

Monocyte phenotype analysis at start of experiment (immediately before treatments/stimulations) and at 24 h after phagocytosis showed the cell positivity for CD14, MHC class II, MHC class I, CD40, and CD87, as well as negativity to apoptosis, as annexin-V-FITC low binding was observed in less than 2% of cells (data not shown). Cell phenotype analysis at day 7 of monocyte differentiation to DC revealed the inhibition of MHC class II and CD 1a (antigen presenting molecules), CD83 (accessory molecule), and CD40 (costimulatory molecule) expression on HZ-fed and 4-HNE-treated DC as compared to unfed control DC (Figure 2), mainly when HZ and 4-HNE were added before GM-CSF/IL-4 stimuli (Figure 2, column “4-HNE before” in histograms). Instead, the expression of MHC class I remained unchanged, and CD87 (uPAR) was 1.9 ± 0.4-fold more expressed in HZ-fed cells as compared to unfed controls.

**Figure 3:**
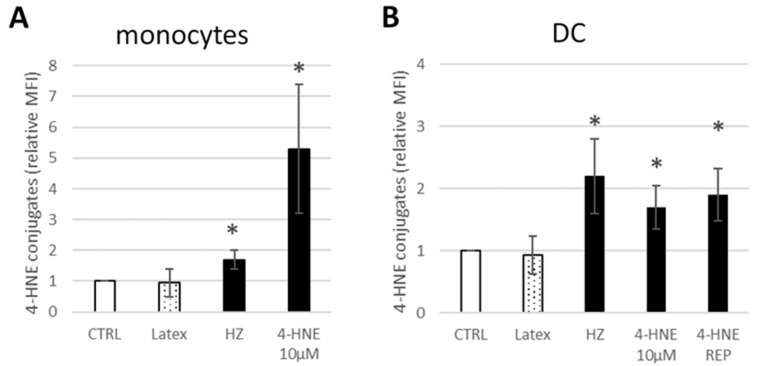
Increased levels of 4-HNE–protein conjugates on the surface of GM-CSF/IL-4-stimuIated, HZ-fed, or 4-HNE-treated monocytes and DC. Untreated control (CTRL), latex bead-fed (Latex) or HZ-fed (HZ) monocytes, or single dose 4-HNE-treated (4-HNE 10 µM) or repeatedly with 1 µM 4-HNE treated (4-HNE REP) monocytes were differentiated to DC during 7 days by stimulation with GM-CSF and IL-4 at 3 h after start of cell incubation and every following 48 h. 4-HNE conjugation was detected on the surface of GM-CSF/IL4-stimulated monocytes at 24 h after start of phagocytosis or 4-HNE treatment (**A**) or on differentiated DC at day 7 of differentiation (**B**) with specific anti-4-HNE-conjugate antibodies and quantified with FITC-labelled secondary antibody by flow cytometry. Fluorescence intensity of bound secondary antibody mirrors the 4-HNE- surface protein conjugation level and is plotted as mean fluorescence intensity (MFI), which was referred to MFI of control cells from the same donor. Means ± SE of 3 independent experiments with monocytes from 3 donors are shown in the figure. The significance of differences (* *p* < 0.01) between controls and HZ- or 4-HNE-treated cells is indicated.

The modified cell phenotype at day 7 was associated with increased levels of 4-HNE-protein conjugates measured on the surface of GM-CSF/IL-4-stimulated HZ-fed or 4-HNE-treated monocytes 24 h after HZ phagocytosis and GM-CSF/IL-4 supplementation and on DC at day 7 of differentiation (Figure 3). Compared to untreated control cells, HZ-fed stimulated monocytes at 24 h and derived DC had 1.7 ± 0.3 and 2.2 ± 0.6 times more 4-HNE adducts on the surface, respectively. Monocytes treated once with 10 µM 4-HNE showed a strong 5.3 ± 2.1-fold increase of 4-HNE-conjugates after 24 h compared to the baseline 4-HNE-conjugation in control monocytes. In DC, after repeated treatment with 1 µM 4-HNE, the 4-HNE-conjugate level on surface was 1.9 ± 0.4 times higher than in controls, while latex feeding did not elicit significant differences from untreated control cells, neither in stimulated monocytes 24 h after phagocytosis nor in DC after 7 days.

### 3.2. HZ and 4-HNE Impaired Functional Binding of Fluorescent GM-CSF

**Figure 4:**
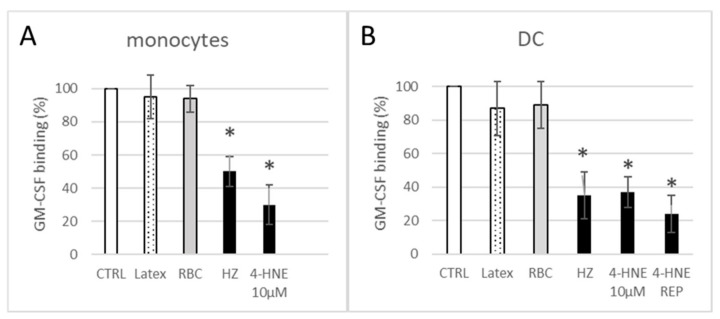
Inhibition of GM-CSF binding in HZ-fed or 4-HNE-treated monocytes and DC. Monocytes were kept as untreated controls (CTRL) or incubated with inert latex beads (Latex), erythrocytes (RBC), HZ, 10 µM 4-HNE as single dose (4-HNE 10 µM), or 1 µM 4-HNE repetitively added every 2 days (4-HNE REP), always before GM-CSF supplementation. Monocytes unstimulated by GM-CSF were analysed for GM-CSF-FITC binding after 24 h (**A**) or were differentiated to DC, and GM-CSF binding assay was performed with DC after 7 days of differentiation (**B**). For assessment of GM-CSF binding, cells were incubated with fluorescent GM-CSF-FITC, washed, and analysed for fluorescence by flow cytometry. Percentage of GM-CSF-FITC binding in treated cells vs. untreated CTRL cells is shown. Absolute values for untreated controls (CTRL) were 29 ± 6 MFI in monocytes and 16 ± 4 MFI in DC. Means ± SE of 3 independent experiments with monocytes from 3 donors are shown. The significance of differences (* *p* < 0.01) between controls and HZ- or 4-HNE-treated cells is indicated.

Due to the impaired GM-CSF/IL-4-dependent differentiation, we hypothesised that the binding capacity of GM-CSF R was affected after HZ phagocytosis. As shown in Figure 4, GM-CSF binding capacity of cells measured as MFI was reduced in HZ-load monocytes and in HZ-load monocyte-derived DC by 50 ± 9% and 65 ± 14%, respectively, as compared to unfed-control cells. Other phagocytosis targets, digestible or not, such as RBCs or latex beads, did not significantly decrease the ligand binding capacity, excluding the non-specific loss of cell membrane by phagocytosis as cause of lower binding levels of GM-CSF after HZ phagocytosis. Interestingly, incubation of cells with 4-HNE recapitulated the significant impairment of GM-CSF-binding seen in HZ-laden cells. A single high-dose bolus of 10 µM 4-HNE decreased the GM-CSF binding by 70% after 24 h, and permanent presence of 1 µM simulated by repeated addition of low-dose 4-HNE each 48 h (4-HNE REP in Figure 4B) resulted in nearly 80% inhibition compared to untreated control DC (Figure 4).

### 3.3. HZ and 4-HNE Dose-Dependently Decreased the Expression of GM-CSF R on Monocyte Surface

**Figure 5:**
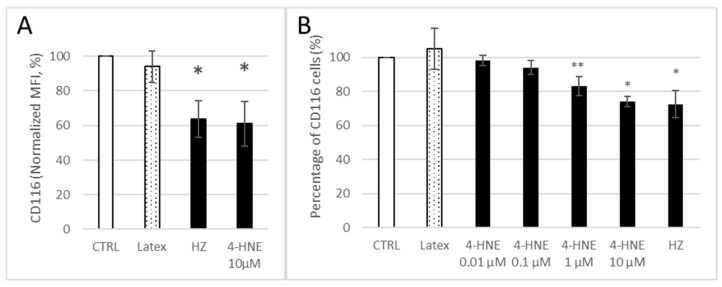
Inhibition of surface expression of CD116 in HZ-fed or 4-HNE-treated monocytes. Monocytes were kept as untreated controls (CTRL), fed with neutral latex beads (Latex) or HZ, or treated with different concentrations of 4-HNE as indicated (**A**,**B**). The surface expression of CD116 was quantified by flow cytometry after immune staining and acquired as MFI (**A**) or percentage of GM-CSF R-positive cells (**B**). Obtained relative MFI and share of CD116-positive treated cells were referred to the respective values measured in untreated control cells from the same donor. Means ± SE of experiments made with monocytes from 3–8 donors. The significances of differences between unfed control and treated cells: ** for *p* < 0.05, * for *p* < 0.01.

To explain the decrease in GM-CSF binding, we evaluated the surface expression of GM-CSF R on HZ-fed and 4-HNE-treated monocytes 24 h after the respective treatment and measured it as MFI (Figure 5A) or share of CD116 positive cells at 24 h after addition of GM-CSF/IL-4 (Figure 5B)**.** CD116 cell surface expression was decreased by 36 ± 11% and 39 ± 13% after HZ phagocytosis or exogenous 4-HNE treatment, respectively (Figure 5A), and the percentage of CD116 expressing cells was decreased by 28 ± 8% in HZ-fed cells and dose dependently in 4-HNE treated cells (Figure 5B). We note that the decrease in CD 116-positive cells (−26 ± 3%) in cells treated with 10 µM 4-HNE is rather similar to the HZ-induced surface GM-CSF-R loss (Figure 5B). The observed effect might have been due to (i) receptor disappearance by aggregation or recycling, (ii) receptor being masked due to re-configuration or aggregation of receptor with another subunit, or (iii) suppressed de novo synthesis.

### 3.4. HZ and 4-HNE Decreased the Expression of GM-CSF R Detected in Cellular Protein Lysate

**Figure 6:**
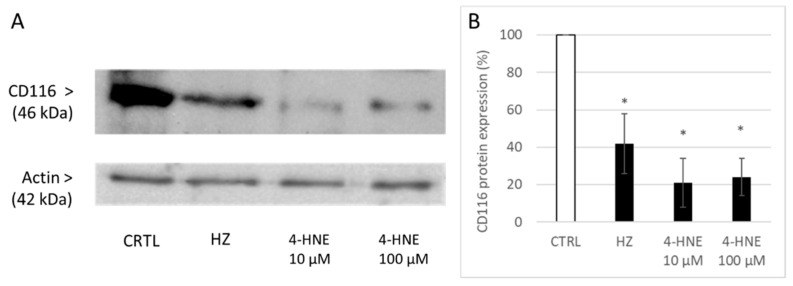
Inhibition of expression of CD116 in whole cell lysate of HZ-fed or 4-HNE-treated monocytes. Protein expression of CD116 was analysed in 20 µg lysate protein from monocytes harvested at day 1 after HZ phagocytosis or 4-HNE treatment. Proteins were separated by one-dimensional SDS-PAGE and Western blotted. Transferred proteins were probed with specific primary anti-CD116 and with anti-actin antibodies, the latter for quality control of transfer and protein load per lane. Bound antibodies were detected by binding of appropriate secondary antibodies and evidenced by ECL. One representative experiment of 3 (**A**) and densitometric analysis (**B**) are shown. Values are referred to controls and expressed as a percentage. Means ± SE of 3 independent experiments from 3 different phagocyte donors are shown. Significant differences are indicated as * *p* < 0.01.

After the observation of surface CD116 reduction, whole cell lysate proteins were checked for GM-CSF R α-chain expression. Decrease of CD116 after HZ by 58 ± 16% and 4-HNE treatment was observed (Figure 6), suggesting an imbalance between degradation and de novo synthesis.

### 3.5. 4-HNE Modified Distinct Amino Acids in GM-CSF R

Given the impaired GM-CSF R functionality (Figure 4) and quantitative decrease of GM-CSF Rα (CD116) expression (Figure 5 and Figure 6) elicited by HZ and 4-HNE, we further investigated qualitative modifications that could affect the GM-CSF R. We performed MALDI analysis of GM-CSF Rα (CD116) protein, extracted from SDS-PAGE. Mascot score for protein identification was higher than 37 in SWISSPROT database searches and higher than 67 in NCBI searches, suggested by Mascot to be significant.

**Figure 7:**
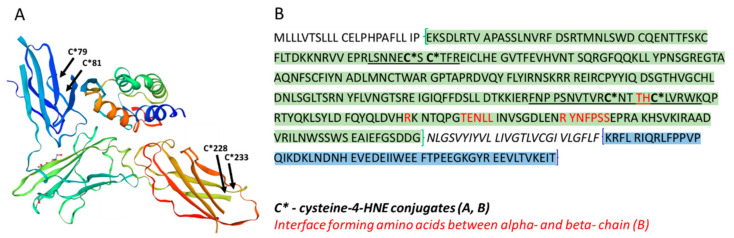
3D structure and amino acid sequence of the alpha-chain of GM-CSF R (CD 116), the positions of amino acids conjugated with 4-HNE, and proximate amino acids essential for correct receptor function. 3D representation of CD116 fragment 35–315 (available online: https://swissmodel.expasy.org/repository/uniprot/P15509?template = 4rs1 (accessed on 29 July 2021)) with indicated 4-HNE conjugated amino acids (**A**). Full amino acid sequence (1–400) of GM-CSF [47] with 4-HNE-conjugated cysteine residues in bold and labelled by * (C*), and underlined 4-HNE-modified peptides detected after trypsin digestion. In red are the interface-forming amino acids between alpha- and beta-chain, described as site 3 [48], the hydrophobic interface with rim of charged residues. Site 3 interacting surface enhanced GM-CSF R binding affinity to GM-CSF. The extracellular domain of receptor is evidenced in green, the intracellular domain in blue (**B**).

In HZ-treated monocytes, we observed the binding of 4-HNE to distinct amino acid residues in CD116 [47]. Posttranscriptional modifications of CD116 by 4-HNE were Michael adducts in the cysteines C79 and C81 from the peptide LSNNECSCTFR (74–84aa), C228 and C233 from the FNPPSNVTVRCNTTHCLVRWK (218–238aa), and CNTTHCLVR (228–236aa). All modifications were located in the extracellular domain (segment 23–320aa) of CD116. Figure 7 illustrates the positions of modified amino acids in the 3D-structure and amino acid sequence of GM-CSF R alpha chain and the localisation of amino acids of known functionally important sites in the receptor.

## 4. Discussion

DC differentiation from human primary monocytes was formerly shown to be strongly impaired by malarial pigment HZ, which is avidly phagocytosed but not digested by the natural precursor cell of DC, in vitro and in vivo [18]. Consequently, HZ persists in the lysosomes of monocytes and catalyses lipid peroxidation, followed by a long-lasting and significant increase of 4-HNE levels in and around HZ-load cells, as shown in [9,21]. Given the difficulty to develop efficient adaptive immunity against malaria, the knowledge of molecular processes behind the impaired differentiation of DC as crucial antigen-presenting cells is essential for efficient prophylactic measures.

The present study confirms the formerly described inhibition of GM-CSF/IL4-dependent ex vivo differentiation of HZ-fed monocytes to DC, as concluded from the inadequate change in expression of functionally relevant surface antigens (Figure 2). The effect of HZ on monocytes was recapitulated by 4-HNE addition either once at high dosage to unfed monocytes (10 µM was the highest 4-HNE concentration compatible with 100% monocyte survival, as reported in [32]) or repeatedly at 1 µM, which are plausible short- and long-term concentrations, respectively, of 4-HNE observed in HZ-fed monocytes. We exclude unspecific phagocytosis-dependent membrane loss as well as persistent lysosome load as the leading cause of decreased surface antigen expression, as differentiation-independent membrane antigens were found unchanged (MHC class I) or even more expressed (CD87) by time and latex-laden monocytes which behaved like unfed controls (Figure 2). Thus, the observed inhibition of GM-CSF/IL-4-dependent differentiation of HZ-loaded cells to DC can be considered a specific HZ-effect and is plausibly mediated by the lipoperoxidation product 4-HNE. The latter assumption is supported by similar impairment of DC differentiation and 4-HNE–protein conjugate levels in 4-HNE-treated monocytes as compared to HZ-fed cells. The stronger inhibition of GM-CSF/IL-4-stimulated DC differentiation by 4-HNE was observed when 4-HNE was supplemented immediately before the cytokines to cells instead of afterwards (Figure 2), suggesting a disturbance in GM-CSF/IL-4 recognition by cells as molecular reason for the HZ and 4-HNE effect on differentiation. This was confirmed by the observation of low GM-CSF binding to DC-precursor monocytes, previously fed with HZ (−50%) or treated with 4-HNE (−70%) compared to untreated control cells (Figure 4). The GM-CSF binding defect appeared to be irreversible as it persisted for 7 days even after a single 4-HNE dose, and less than 30% of cytokine were bound to HZ-containing, once or repeatedly with 4-HNE-treated DC as compared to untreated controls. GM-CSF binds preferentially to the GM-CSF R, which is expressed in hematopoietic cells, in monocytes and DC [48]. The surface expression of the receptor is low, with 100–1000 copies per cell [39]. In the study presented here, phagocytosis of HZ but not inert latex beads downregulated the expression of the GM-CSF-specific alpha subunit of the receptor (CD116) in monocytes after one day (Figure 5A) by approximately 40%, and HZ completely erased CD116 from the membrane in 30% monocytes (Figure 5B). The partial or complete disappearance of the receptor from vital HZ-fed monocytes may result from enhanced shedding of the receptor from the cell surface or an insufficient de novo synthesis of GM-CSF R or, more likely, a combination of both. Both processes might be explained by the elevated level of lipoperoxidation and its secondary products, 4-HNE and 15-hydroxyeicosatetraenoic acid (15-HETE), in monocytes that have phagocytosed HZ. In fact, 4-HNE was able to recapitulate the decrease of CD116 surface expression by HZ in monocytes in a dose-dependent manner (Figure 5). Firstly, 4-HNE and 15-HETE induce and activate as physiologic ligand the nuclear transcription factor PPAR-gamma [49], which is able to interfere with the master regulator PU.1 and the transcription factor NF-kappa-B for GM-CSF R [32,50]. The interference with PU.1 and trans-repression of nF-kappa-B by activated PPAR-gamma may explain the downregulation of CD116 expression and halt DC differentiation in the presence of HZ and 4-HNE observed in this study. Secondly, HZ phagocytosis and 15-HETE may elicit shedding of GM-CSF R from monocyte membranes [51] by activating metalloproteases in human monocytes, as described in [52]. From performed analysis we cannot conclude yet whether the inhibition of receptor de novo synthesis or the activation of receptor degradation is the prior mechanism by which HZ and lipoperoxidation products downregulate CD 116, although the decrease of total cellular CD116, which was measured in lysate and exceeds the surface loss, may favour the synthesis. Further studies are needed to confirm this hypothesis.

Lipoperoxidation as cause for downregulation of CD116, as observed during initial pro-inflammatory immune cell response to malaria product HZ, may not be restricted to malaria but may also underlay other inflammatory pathologies, accompanied by a decreased receptor expression such as chronic alcohol intoxication [53] or inflammatory bowel disease [54].

Finally, 4-HNE affected not just the expression/degradation but also modified the protein structure of GM-CSF R in HZ-fed or 4-HNE-treated monocytes. The highly reactive aldehyde formed adducts with four cysteine residues of the CD116 alpha chain of the receptor, two of them, the cysteins in position 228 and 233, in close proximity to amino acids threonine 231 and histidine232, which are crucially engaged in alpha-beta-chain interface formation [48]. Figure 7 illustrates the sites of posttranslational modification by 4-HNE embedded in the 3D structure and amino acid primary structure of GM-CSF R alpha-chain (CD116) and the localisation of amino acid residues that form the interface with the beta-chain. The interaction between alpha-chain (CD116) and the beta-subunit is the prerequisite for high affinity binding of GM-CSF, and modifications of the hydrophobic interface should impact on receptor function. The introduction of two hydrophobic 9 carbon atom long moieties and two aldehyde groups by 4-HNE adduction nearest to the alpha-beta-chain interface may plausibly disturb the subunit interaction, but this needs to be confirmed experimentally. Whether the other two observed modifications have a functional impact on the receptor function remains unrevealed.

The introduction of 4-HNE adducts in the nascent protein chain during translation and export may also impact on CD 116 export to the membrane, thus resulting in decreased expression and enhanced degradation, a commonly observed event for 4-HNE tagged proteins [55]. GM-CSF has been reported to increase during malaria infection [56,57]. The cytokine is essential for differentiation of hematopoietic stem cells to erythrocytes, neutrophils, monocytes, eosinophils, and megakaryocytes [58] and its priming activity on terminally differentiated myeloid cells is well recognised [58,59] and the rationale for inclusion of one plasmid DNA encoding human GM-CSF in a multivalent DNA malaria vaccine [60]. In contrast, there are no studies on GM-CSF R expression in malaria that allow final conclusions on receptor changes in disease. Only one study has reported unchanged receptor expression in patients with uncomplicated malaria, determined, however, in frozen and stored granulocyte lysates [61]. Further studies in malaria patients have the potential to reveal whether granulocytes behave differently to monocytes in malaria infection regarding their GM-CSF R expression and whether the herein described 4-HNE-dependent changes of GM-CSF R are recapitulated in malaria patients.

In summary, HZ and HZ-derived lipoperoxidation product 4-HNE profoundly interfere with the expression, structure, and function of GM-CSF R in human DC precursor cells, resulting in the impairment of GM-CSF/IL4-dependent DC differentiation in vitro. Interference of 4-HNE with immune cells may also explain the inhibitory effects on peripheral blood DC observed in children with acute *Pf* malaria [2], and thus the incomplete and short-lasting immunity mounted to malaria infection. Oxidative stress and lipoperoxidation are hallmarks of infectious and inflammatory diseases. Derived 4-HNE can act as potent modulator of immune cell differentiation and function by modifying the crucial receptor in hematopoiesis and immune control, the GM-CSF R.

## 5. Conclusions

Malaria pigment HZ is known to strongly impair several immune responses such as DC differentiation. Oxidative stress and lipoperoxidation are hallmarks of malaria and other infectious and inflammatory diseases. HZ and lipoperoxidation-derived 4-HNE are shown here to modify the cellular GM-CSF R expression and provoke posttranslational modifications of the receptor, which both may explain the impaired DC differentiation in HZ-laden or 4-HNE-treated precursor monocytes. The observed modifications of GM-CSF R by lipoperoxidation products may contribute to gain insight into the inadequate immune response described in malaria but may also play a role in other inflammatory diseases.

## Data Availability

Data is contained within the article.
